# Transsternal Approach for Broncho-Pleural Fistula Closure After Right Pneumonectomy

**DOI:** 10.7759/cureus.50397

**Published:** 2023-12-12

**Authors:** Andrei I Gritsiuta, Charles T Bakhos, Abbas E Abbas, Roman V Petrov

**Affiliations:** 1 Department of Surgery, University of Pittsburgh Medical Center, Pittsburgh, USA; 2 Department of Thoracic Medicine and Surgery, Lewis Katz School of Medicine at Temple University, Philadelphia, USA; 3 Department of Thoracic Oncology, Warren Alpert School of Medicine at Brown University, Providence, USA; 4 Division of Cardiothoracic Surgery, University of Texas Medical Branch John Sealy School of Medicine, Galveston, USA

**Keywords:** post pneumonectomy, sternotomy, transsternal bronchial occlusion, broncho-pleural fistula, pleural empyema

## Abstract

Broncho-pleural fistula (BPF) is an abnormal communication between the bronchial lumen and the pleural space that typically occurs postoperatively. Surgical intervention is typically needed to patch the fistula; however, current literature lacks a gold standard for which treatment to use. With a high mortality rate, there is a clear urgency for quick and successful intervention. This case examines a 59-year-old patient presenting with a BPF 14 years after incidental pneumonectomy during upper lobectomy for invasive aspergillus. A fistula was appreciated during bronchoscopy with contrast injection. The fistula was closed via the transsternal approach through median sternotomy and pericardiotomy. This case report aims to provide a viable option to successfully repair a BPF via the transsternal approach.

## Introduction

Broncho-pleural fistula (BPF) is an abnormal communication between the bronchial lumen and pleural space [1]. Typically, a BPF forms within the first two weeks ensuing anatomic lung resections, and the incidence depends on the resection type [2]. BPF formation following a lobectomy is up to 1% incidence, whereas it is 4.5% to 20% following a pneumonectomy [3]. Clinical presentation manifests with dyspnea, chest pain, cough with sputum, subcutaneous emphysema, and sepsis. The presence of new or increased pleural air on imagining confirms the diagnosis of fistula formation [4]. Management of BPF is challenging and surgical intervention is typically required. Mortality rates vary from 18% to 67% creating an urgency for expedient workup and intervention [5]. Although there are multiple intervention possibilities for BPF management, there is no current consensus due to over complexity of the condition and individual patient factors. Treatment options vary significantly from a multitude of surgical interventions to alternative treatments such as endoscopic stump closure with an autologous blood patch, argon plasma coagulation, cryoprecipitate glue, vacuum-assisted closure, and Fuji Systems® Uniblocker (Ambu Inc, Columbia, Maryland, United States) [6]. This case report aims to describe a treatment option for BPF following a pneumonotomy via transsternal bronchial occlusion.

## Case presentation

**Figure 1:**
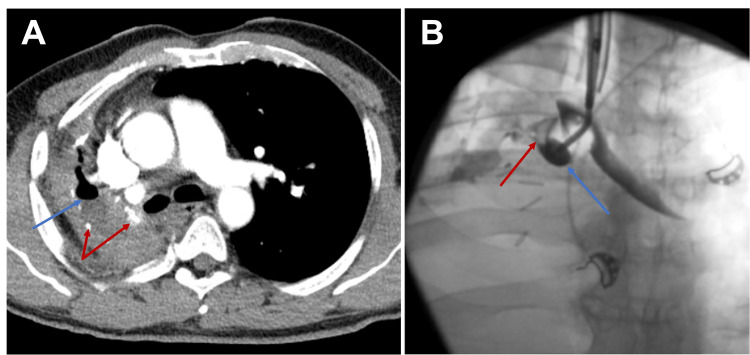
Preoperative evaluation: (A) Chest CT scan of the 59-year-old male showing calcifications (red arrows) and air-fluid (blue arrow) levels within the right post-pneumonectomy residual pleural cavity; (B) Contrast extravasation to the residual pleural space (red arrow) injected through the bronchial blocker in the right bronchial stump (blue arrow) confirming the presence of a broncho-pleural fistula.

A 59-year-old male patient presented with intermittent productive cough, dyspnea on exertion, and recent syncopal episodes. He had a complex past medical history, including right incidental pneumonectomy due to pulmonary artery injury during upper lobectomy for invasive aspergillosis 14 years prior at an outside facility. At this time, chest CT demonstrated calcifications and air-fluid level within the right post-pneumonectomy residual pleural cavity (Figure 1A). Given his past medical history and presentation, there was a concern for BPF; however, no direct communication between the pleural space and the bronchial stump was noted on the initial bronchoscopy. The total length of the bronchial stump was measured at 3 cm. BPF was confirmed during subsequent bronchoscopy by injecting 20 ml of contrast through a bronchial blocker, a thin semi-rigid plastic catheter with a high-volume low-pressure balloon at its distal tip, under fluoroscopic guidance (Figure 1B).

**Figure 2:**
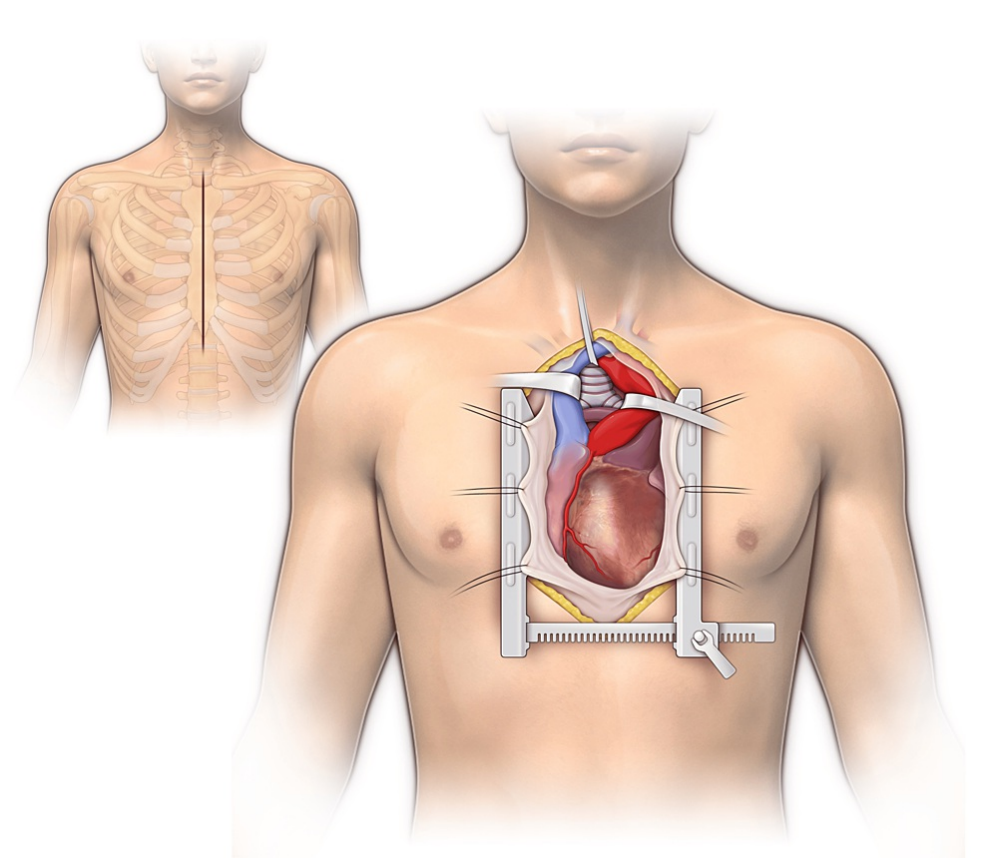
Transsternal transpericardial approach to the right bronchial stump for broncho-pleural fistula occlusion. Figure Credit: Author Roman V. Petrov

After drainage and sterilization of the residual cavity with a pigtail catheter and a course of antibiotic therapy, the patient was brought to the operating room for transsternal bronchial stump occlusion. The procedure was performed through median sternotomy and pericardiotomy (Figure 2).

An elongated right pulmonary artery stump was trimmed in order to achieve better visualization during the operation. The right bronchial stump was divided with TA 30 blue staple load (Medtronic, Minneapolis, Minnesota, United States) under bronchoscopy control to avoid narrowing of the lumen. The vascularized pedicled soft tissue flap of the pericardium and thymus was harvested and used for bronchial stump reinforcement. The technical aspects of the transsternal occlusion of the right main stem bronchial stump are shown in Video 1. The patient had an uneventful postoperative course. He was extubated on postoperative day one, had drains removed on postoperative day four, and was discharged home in stable condition on postoperative day five. The patient remained asymptomatic without BPF recurrence four years postoperatively.

**Video 1:** A demonstration of the technical aspects of the transsternal occlusion of the right main stem bronchial stump in the treatment of chronic empyema with broncho-pleural fistula after pneumonectomy. The video has been previously presented at the 103rd American Association for Thoracic Surgery (AATS) Annual Meeting and published in the AATS video library.

## Discussion

BPF is a relatively uncommon postoperative complication that has serious consequences if not corrected urgently. Patients presenting post anatomic lung resection should raise high suspicion for BPF when sudden onset of chest pain, dyspnea, and hemodynamic instability presents within the first two weeks. Once the proper diagnosis is established, surgical intervention should be considered in non-septic patients. Current literature lacks a clear gold standard surgical treatment option for BPF correction. In the case presented, a transsternal occlusion of the main bronchial stump was utilized. In our opinion, it is the most effective treatment option available for patients with a long bronchial stump. Transsternal occlusion provides a direct access to the distal tracheal and carina, avoids potential infected pleural space, facilitates surgical closure, and reconstructs the airway. Such, analysis of transsternal occlusion during a nearly forty-year timeline showed an overall 80.8% success rate [7]. This analysis concluded that postoperative complications occurred in only 17% of patients, thus, showing the effectiveness that transsternal occlusion has on correcting BPF. Ginsberg and Saborio analyzed six case series including 116 transsternal occlusions of BPF with 0-30% recurrency rate. These authors concluded that transsternal occlusion of the main bronchial stump is preferable in patients with recurrent BPF, any contraindications to thoracotomy, or when concomitant carinal resection is required [8]. In another case series, 13 patients underwent a transsternal approach for a bronchial stump repair. Of these 13 patients, only one (7.6%) patient had BPF recurrence [9]. When compared to a transthoracic approach with tissue flap transposition, transsternal occlusion demonstrated to be a more viable option with treatment success rate of 92% [10].

## Conclusions

Our report demonstrates the continued utility of the transsternal approach for the closure of the BPF. We find that the transsternal occlusion of a bronchial stump is easier and more reproducible than the transthoracic approach with tissue flap transposition in patients with chronic pleural empyema with BPF and long bronchial stump, due to less morbidity, favorable outcomes, and good reproducibility.
